# Antibiogram profiles of pathogenic and commensal bacteria in goat and sheep feces on smallholder farm

**DOI:** 10.3389/frabi.2024.1351725

**Published:** 2024-05-16

**Authors:** Ashesh Basnet, Agnes Kilonzo-Nthenge

**Affiliations:** Department of Food and Animal Sciences, Tennessee State University, Nashville, TN, United States

**Keywords:** antibiotics resistance, goats and sheep, small-scale farms, foodborne pathogens, *Salmonella*, *E. coli*, *Stapyhlococcus aureus*, *Shigella*

## Abstract

**Introduction:**

The increase of antimicrobial resistance (AMR) in zoonotic pathogens poses a substantial threat to both animal production and human health. Although large-scale animal farms are acknowledged as major reservoirs for AMR, there is a notable knowledge gap concerning AMR in small-scale farms. This study seeks to address this gap by collecting and analyzing 137 fecal samples from goat and sheep farms in Tennessee and Georgia.

**Method:**

Bacteria were identified using culture-dependent methods and polymerase chain reaction (PCR), and antimicrobial susceptibility testing (AST) was performed using the Kirby-Bauer Disk Diffusion method.

**Results and discussion:**

The prevalence of *E. coli* (94.9%) in goats and sheep significantly exceeded (p < 0.05) that of *S. aureus* (81.0%), *Shigella* (35.0%), *S. saprophyticus*, and *Salmonella* (3.0%). *Salmonella* occurrence in goat feces (2.2%) was higher than in sheep (0.8%). Notably, 27% of goats and 8% of sheep tested positive for *Shigella* spp., while 60% of goats and 21% of sheep tested positive for *S. aureus.* Antibiotic resistance was observed primarily against ampicillin (79.4%), vancomycin (65.1%), and gentamycin (63.6%), significantly surpassing (p < 0.05) resistance to tetracycline (41.6%) and imipenem (21.8%). The penicillin (79.4%), glycopeptide (65.1%), and aminoglycoside (63.6%) antibiotic classes displayed significantly higher (p < 0.05) resistance compared to tetracyclines (45.7%) and carbapenem (21.8%). Our findings suggest that goats and sheep feces may serve as source for multidrug-resistant bacteria, raising concerns about the potential introduction of their fecal matter into soil, water, and eventually to the food chain. This highlights the need for proactive measures to address and mitigate AMR in goats and sheep within small-scale farms.

## 1 Introduction

Ruminants are one of the most affordable farm animals to raise for self-sufficiency and revenue. Goats and sheep easily adapt to a wide range of climates (Koluman Darcan and Silanikove, 2018) and require less effort than other farm animals as they are low maintenance. These animals require the least housing spaces, minimal maintenance costs, small investment, and low-cost production expenses. Beyond their cost-effectiveness, these ruminants are gaining popularity for providing milk, meat, wool, hide, and fiber. Milk and meat from sheep and goats are highly nutritious and easily digestible (Cordeiro et al., 2022), essential for individuals with certain digestive sensitivities or allergies. Furthermore, the United States of America (USA) is experiencing a growing demand for goat and sheep meat, driven in part by the settlement of diverse ethnic populations. The influx of African, Asian, and Hispanic communities to the USA has played a significant role in popularizing ethnic cuisines, influencing the broader American food culture (Hart et al., 2019).

Goat and sheep farming face obstacles hindering efficient and cost-effective production, including limited understanding of diseases, and inadequate access to veterinary services (Abdulai, 2022). Amoxycillin, ampicillin, gentamicin, florfenicol, oxytetracycline, penicillin, and tylosin are some of the major antibiotics used to treat diseases and infections in goats and sheep (Lianou and Fthenakis, 2022). However, the exposure of both commensal and pathogenic microbes to antibiotics could lead to the development of antimicrobial resistance (Briers et al., 2014). Antimicrobial resistance is a pressing global public health threat and also contribute to severe economic loss to the goat and sheep farmers.

In goat and sheep production, notable pathogenic bacteria, including *Staphylococcus aureus, Staphylococcus saprophyticus, Escherichia coli*, and *Salmonella* spp., pose a significant health risk (Hanlon et al., 2018; Ariffin et al., 2019; Andrade et al., 2021). The presence of S. *aureus* in goat products, documented by Aqib et al. (2019) and Angelidis et al. (2020), represents a significant threat to public health. Furthermore, numerous outbreaks associated with *Salmonella* spp. have been linked to the consumption of contaminated goat products, as highlighted by Robinson et al. (2020). Jacob et al. (2013) found an 11.1% prevalence of *E. coli* O157:H7 in goat feces at slaughter, suggesting a potential for contamination of goat meat during slaughtering. Given the documented antibiotic resistance in bacterial strains and the potential role of widespread antibiotic use in the emergence of antimicrobial resistance (AMR), it is imperative to evaluate AMR in small-scale goat and sheep farms, especially considering the zoonotic nature of several diseases in these animals. Farms with average 231 acres or less are classified as small farms by the U.S. Department of Agriculture (USDA). Evaluating antimicrobial resistance (AMR) in small-scale goat and sheep farms is crucial, especially given the growing demand for goat and sheep meat dishes in several states across the USA. Notably, states such as Tennessee (TN) and Georgia (GA) are experiencing an upswing in demand, driven partly by immigration patterns, and shifting demographics. Notably, existing research in livestock has predominantly centered on larger food animal farms, creating a substantial knowledge gap in comprehending AMR dynamics in smaller, traditional farming settings. This study aims to fill this void by examining the prevalence of pathogenic and commensal bacteria, along with AMR profiles, in small-scale goat and sheep farms in Tennessee and Georgia, USA, given the limited studies on AMR in ruminants.

## 2 Materials and methods

### 2.1 Farm visitation and sample collection

The study received approval from the Institutional Animal Care and Use Committee (IACUC; #TSU21-04) at Tennessee State University (TSU), ensuring compliance with animal welfare and protection standards. Fecal samples were collected from a total of 30 farms, comprising 17 goat and 7 sheep farms in Tennessee, as well as 6 goat farms in Georgia. A sample size of 137 ruminants (104 goats and 33 sheep) was investigated, with fecal samples randomly obtained from 10% of the total herd size of each farm, following the methodology recommended by Amin et al. (2022). In Tennessee, farms were located across 14 counties (Marshall, Giles, Bedford, Lincoln, Franklin, Rutherford, Davidson, Unicoi, Claiborne, Madison, Maury, Moore, Overton, Hawkins), while in Georgia, they were distributed across six counties (Pulaski, Barrow, Effingham, Brooks, Houston, Daughtry). Fecal samples from goats and sheep were collected using a modified Bauman et al. (2016) method, where 4–6 grams were obtained from each animal’s rectum by the same handler and placed in individual sterile plastic vials. Disposable polyethylene gloves, lubricated with 0.3 mL of sterile OB Lube Non-Spermicidal Sterile Lubricating Jelly (VetOne, Boise, Idaho, USA), were changed for each animal to prevent cross-contamination. Each sample was labeled with the corresponding farm identification number and promptly transported in a cooler with ice packs to the Food Microbiology and Safety Laboratory at TSU. Samples collected from Tennessee were stored at −20°C for approximately one week before undergoing microbial analysis. Conversely, samples from Georgia farms were shipped overnight to the laboratory, frozen at −80°C due to the high work volume at the laboratory and processed within a timeframe of 7–21 days.

### 2.2 Pre-enrichment of fecal samples and detection of pathogens

The frozen rectal samples were thawed at room temperature. Approximately 2 grams of each sample were weighed and homogenized with 18 ml of Buffered Peptone Water (BPW) (CM0509; Oxoid, Basingstoke, UK) in a sterile stomacher bag (Fisher Scientific, Waltham, Massachusetts, USA). Homogenization was conducted using the 400 Circulator Stomacher^®^ (Seward, Norfolk, UK) at 230 rpm for 1 minute. Subsequently, 10 ml of each homogenized sample was incubated at 37°C for 24 hours. This incubation was performed to test for the presence of *E. coli, S. aureus, S. saprophyticus, Salmonella* spp., and *Shigella* spp. in goat and sheep feces.

### 2.3 Microbial analysis and bacterial identification

#### 2.3.1 Detection of *Escherichia coli*

From each pre-enriched sample, 1 ml of the pre-enriched solution was subjected to 9 ml of Tryptic Soy Broth (TSB) (BD, Franklin Lakes, New Jersey, USA) at 37°C for 24 hours. After incubation for 24 hours, 10 µl of loop sample were streaked in triplicates onto Eosin-Methylene Blue (EMB) agar plates (BD, Franklin Lakes, New Jersey, USA). Presumptive *E. coli* isolates exhibiting a characteristic morphology of blue/black with a greenish metallic sheen on EMB were stored in 80% glycerol at a −80°C freezer for further analysis.

#### 2.3.2 Detection of *Staphylococcus aureus*

From the pre-enriched rectal sample, 1 ml of the enriched solution was subjected to 9 ml of Tryptic Soy Broth (TSB) (BD, Franklin Lakes, New Jersey, USA) at 37°C for 24 hours. After incubation for 24 hours, 10 µl of loop sample were streaked in triplicates onto CHROMAgar *Staph aureus* agar plates (BD, Franklin Lakes, New Jersey, USA). Presumptive *S. aureus* isolates exhibiting a characteristic pink color were stored in 80% glycerol at a −80°C freezer for further analysis.

#### 2.3.3 Detection of *Staphylococcus saprophyticus*

From each pre-enriched sample, 1 ml of the enriched solution was subjected to 9 ml of Tryptic Soy Broth (TSB) (BD, Franklin Lakes, New Jersey, USA) at 37°C for 24 hours. After incubation for 24 hours, 10 µl of loop sample were streaked in triplicates onto selective media- CHROMAgar *Staph aureus* agar plates (BD, Franklin Lakes, New Jersey, USA). The agar plates were incubated at 37°C for 24 hours. Presumptive *S. saprophyticus* isolates characterized by the appearance of blue colonies on the CHROMAgar were preserved in 80% glycerol at a −80° for further analysis.

#### 2.3.4 Detection of *Salmonella* spp.

From each of the pre-enriched samples, 1 ml of the enriched solution was subjected to 9 ml of Tetrathionate (TT broth) (BD, Franklin Lakes, New Jersey, USA) at 37°C for 24 hours. After incubation for 24 hours, 10 µl of loop sample were streaked in triplicates onto selective media-Xylose Lysine Deoxycholate agar plates (XLD) (BD, Franklin Lakes, New Jersey, USA) and thereafter incubated at 37°C for 24 hours. Although XLD agar selects only H_2_S *Salmonella* producers, there could be H_2_S-negative *Salmonella* strains that could be present in the goat and sheep feces. Presumptive *Salmonella* spp. isolates exhibiting a characteristic dull yellow and with a black center were stored in 80% glycerol at −80° for further analysis.

#### 2.3.5 Detection of *Shigella* spp.

Each enriched sample, comprising approximately 1 ml, was mixed with 9 ml of Tetrathionate (TT broth) (BD, Franklin Lakes, New Jersey, USA) and incubated at 37°C for 24 hours. Following incubation, 10 µl were streaked in triplicates onto selective media‐Xylose Lysine Deoxycholate agar plates (XLD) (BD, Franklin Lakes, New Jersey, USA). Presumptive *Shigella* spp. isolates, characterized by a dull yellow color, were stored in 80% glycerol at −80° for further analysis.

### 2.4 DNA extraction of presumptive isolates

Presumptive *E. coli, S. aureus, S. saprophyticus, Salmonella* spp., and *Shigella* spp. isolates, previously stored at −80°C, were thawed at room temperature and thereafter transferred to TSB broth and incubated overnight at 37°C. Following incubation, DNA was isolated from these cultures using the Qiagen DNeasy^®^ UltraClean^®^ Microbial Kit (QIAGEN, Hilden, Germany) as per the manufacturer’s instructions. Approximately 2 ml of culture was extracted from each sample, and ultimately, 100 µl of DNA was eluted. The DNA concentration of each sample was determined using the Qubit 1x High Sensitivity dsDNA assay (Invitrogen, Waltham, Massachusetts, USA).

### 2.5 PCR confirmation of presumptive isolates

Molecular confirmation of all presumptive bacterial isolates was conducted using PCR. The PCR mix was prepared with Qiagen HotStar Taq Polymerase (QIAGEN, Hilden, Germany) following the manufacturer’s instructions. DNA concentrations were assessed, and approximately 100 ng/ml of DNA was utilized per reaction. The PCR mixture was composed of the following components: 10µl of 2X HotStar Taq Plus Master Mix, 0.5µM of both forward and reverse primers, 2µl of 10X CoralLoad Concentrate dye, 100ng of template DNA, and RNase-free water was added to achieve a final reaction volume of 20µl. The sequences of primer pair used for targeting *Escherichia coli* target gene (16Sr RNA) was 5′-AGAGTTTGATCATGGCTCAG-3 and 5′-GGACTACCAGGGTATCTAAT-3′ (Mamun et al., 2016), whereas the primer pair used for targeting *Salmonella* spp. (*sdi*A) was 5′-CGGTGGTTTTAAGCGTACTCTT-3′ and 5′-CGAATATGCTCCACAAGGTTA-3′ (Halatsi et al., 2006). *Shigella* spp (*vir*A) primer pair was 5-CTGCATTCTGGCAATCTCTTCACATC-3′ and 5′-TGATGAGCTAACTTCGTAAGCCCTCC-3′ (Villalobo and Torres, 1998), for *S saprophyticus* (16Sr RNA) the primer pair was 5′-T TCAAAAAGTTTTCTAAAAAATTTAC-3′ and 5′-ACGGGCGTCCACAAAATCAATAGGA-3′ (Martineau et al., 2000), and *S. aureus* (16SrRNA) was 5′-CCTATAAGACTGGGATAACTTCGGG-3’ and 5′-CTTTGAGTTTCAACCTTGCGGTCG-3′ (Mason et al., 2001). A Generural 100bp ladder DNA ladder (Thermoscientific, Massachusetts, USA) was used as a marker. PCR was performed by using a GeneAmp PCR system 2700 thermal cycler (Applied Biosystems, California, USA). Cycling parameters for PCR amplification of pathogenic and commensal bacteria are shown in **Table 1**. Consequently, the PCR products were electrophoresed in agarose gel stained with 0.5 µg/mL of (VWR, Radnor, Pennsylvania, USA) and thereafter viewed under Bio-Doc Gel Doc Ez Imager Documentation Imaging System (BioRad, Hercules, California, USA).

**Table 1:** Primer sequences, target gene and amplification size for selected bacteria species.

Target bacteria	Primer Sequence (5’–3’)	Target gene	Amplified segment (bp)	Reference
*Escherichia* *coli* spp.	Fwd: AGAGTTTGATCATGGCTCAG Rev: GGACTACCAGGGTATCTAAT	16Sr RNA	585	Mamun et al., 2016
*Salmonella* spp.	Fwd: CGGTGGTTTTAAGCGTACTCTT Rev: CGAATATGCTCCACAAGGTTA	*sdi*A	274	Halatsi et al., 2006
*Shigella* spp.	Fwd: CTGCATTCTGGCAATCTCTTCACATC Rev: TGATGAGCTAACTTCGTAAGCCCTCC	*vir*A	215	Villalobo and Torres, 1998
*Staphylococcus* *saprophyticus*	Fwd: TCAAAAAGTTTTCTAAAAAATTTAC Rev: ACGGGCGTCCACAAAATCAATAGGA	16S rRNA	186	Martineau et al., 2000
*Staphylococcus* *aureus*	Fwd: CCTATAAGACTGGGATAACTTCGGG Rev: CTTTGAGTTTCAACCTTGCGGTCG	16Sr RNA	798	Mason et al., 2001

### 2.6 Antibiotic susceptibility testing

**Figure 1:**
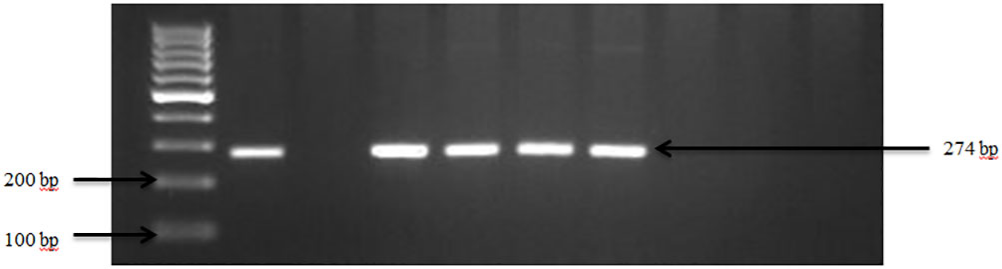
PCR amplification of the *sdi*A gene in *Salmonella* spp. Lane 1: 100bp Ladder, Lane 2: Positive control *Salmonella typhimurium* ATCC 14028, Lane 3: Negative control *E. coli*, Lane 4–7: Samples.

**Figure 2:**
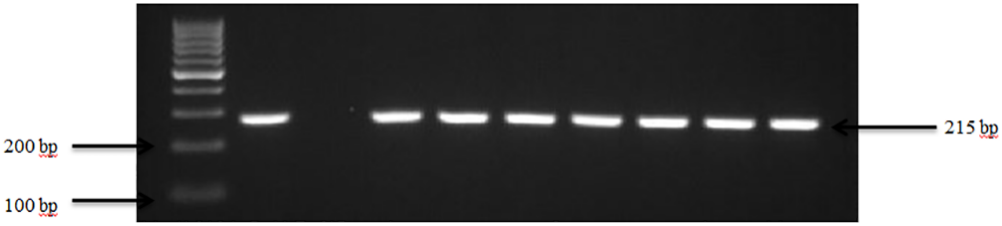
PCR amplification of the *vir*A in *Shigella* isolates. Lane 1: 100bp Ladder, Lane 2: Positive control *Shigella dysenteriae* ATCC 13313, Lane 3: Negative control *E. coli*, Lane 4-10: Samples.

**Figure 3:**
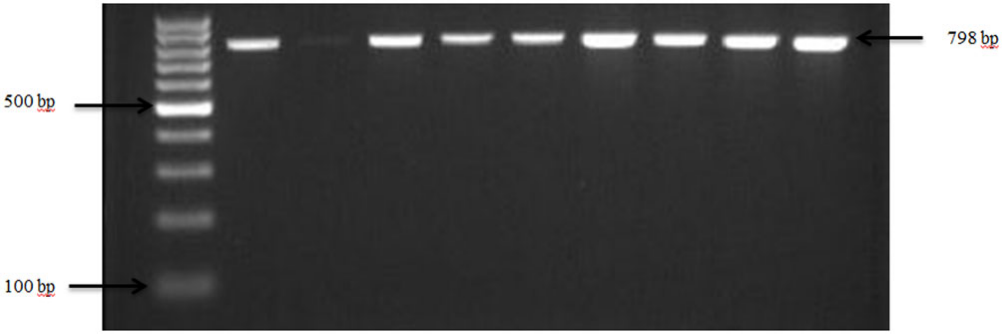
PCR amplification of the 16Sr RNA gene in *S. aureus* isolates. Lane 1: 100bp Ladder, Lane 2: Positive control *S. aureus* ATCC 25923, Lane 3: Negative control *E. coli*, Lane 4–10: Samples.

**Figure 4:**
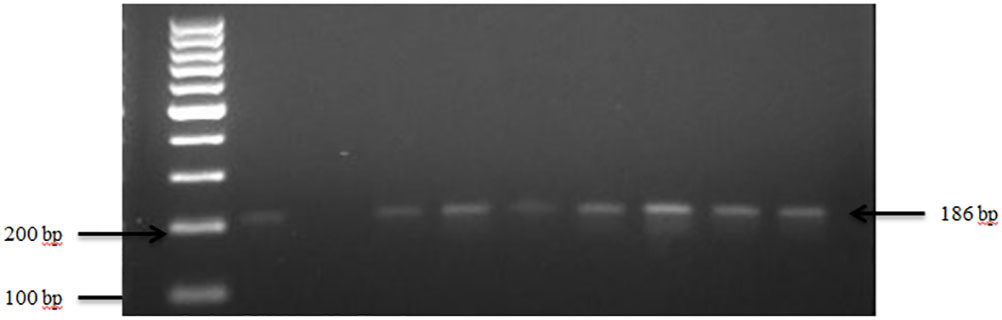
PCR amplification of the 16Sr RNA gene in *S. saprophyticus* isolates. Lane 1: 100bp Ladder, Lane 2: Positive control *S. saprophyticus* ATCC 49907, Lane 3: Negative control *E. coli*, Lane 4–10: Samples.

**Figure 5:**
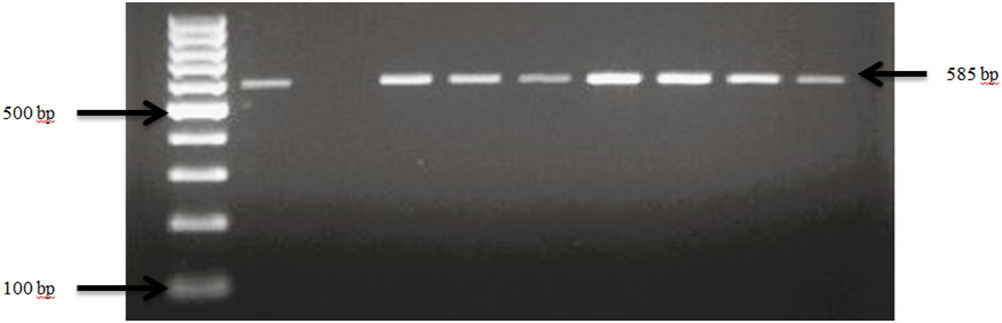
PCR amplification of the 16Sr RNA gene in *E. coli* isolates. Lane 1: 100bp Ladder, Lane 2: Positive control *E. coli* ATCC 25922, Lane 3: Negative control *Salmonella typhimurium* ATCC 14028, Lane 4–10: Samples.

Presumptive pathogenic bacteria isolates identified by culture method were confirmed by PCR as described in Materials and Methods. Our results showed that *sdi*A and *vir*A genes were amplified for *Salmonella* spp. and, *Shigella* spp. whereas 16S rRNA genes were amplified for *S. aureus*, *E. coli*, and *S. saprophyticus* (**Figures 1**, **2**, **3**, **4**, **5**). Antibiotic Susceptibility Testing (AST) was conducted using the standard Kirby-Bauer disk diffusion method, following the recommendations of the Clinical and Laboratory Standards Institute (CLSI). An inoculum from each selected bacterial culture was mixed in 5 ml of TSB and thereafter inoculated on Muller-Hinton agar (MHA) plates (CM0337B; Oxoid, Hampshire, England). The turbidity of the culture was adjusted to 0.5 McFarland before plating on MHA. The antibiotic discs (Oxoid, Hampshire, United Kingdom), along with their concentrations in parentheses, included ampicillin (10 µg), imipenem (10 µg), cephalothin (30 µg), cefpodoxime (30 µg), vancomycin (30 µg), gentamicin (15 µg), doxycycline (30 µg), ceftiofur (30 µg), florfenicol (30 µg), tetracycline (30 µg), and oxytetracycline (30 µg). Recognizing their public health significance, cephalosporins (cephalothin, cefpodoxime), fluoroquinolones (imipenem), and vancomycin, which find use in human medicine, were included for assessment. Such monitoring aids in evaluating the potential transmission risk of resistant bacteria from animals to humans via the food chain or direct contact. Moreover, ampicillin and gentamicin have historically been utilized for clinical mastitis treatment in goats and sheep across various regions, including Greece (Lianou and Fthenakis, 2022). Similarly, tetracyclines like doxycycline, oxytetracycline, and tetracycline are commonplace in goat and sheep production (Ibrahim et al., 2020). The selection of Imipenem and florfenicol for this study was informed by prior antimicrobial susceptibility testing studies (Rule et al., 2004; Banerjee et al., 2022). Florfenicol, a broad-spectrum antibiotic commonly employed in the U.S. for treating respiratory and enteric infections in goats, was chosen based on its efficacy (Wu et al., 2023). A disc dispenser (Oxoid, Solon, OH, USA) was employed to place antibiotic discs on MHA plates heavily inoculated with bacterial cultures, and the plates were then incubated at 37°C for 24 hours. Four discs were dispensed per MHA plate with proper spacing. Inhibition zones were recorded by measuring the diameter, and results were interpreted as susceptibility, intermediate, and resistance according to CLSI (2020). *Staphylococcus aureus* ATCC 25923 and *Escherichia coli* ATCC 25922 were used as quality control organisms. The Food and Drug Administration diffusion procedure recommends these Seattle strains as stable reference standard bacterial strains for AST (Coyle et al., 1976). The reference bacteria were tested concurrently with the isolated bacteria as controls.

### 2.7 Data analysis

All data were transferred to a Microsoft Excel 2021 spreadsheet (Microsoft Corp., Redmond, WA, USA) for analysis. The prevalence and antibiotic resistance values were expressed as percentages. The prevalence and antibiotic resistance data were analyzed using the analysis of variance of SAS for Windows (SAS Institute, Inc., North Carolina, USA), incorporating both analysis of variance and chi-square tests. P values of less than 0.05 were considered statistically significant.

## 3 Results

### 3.1 Pathogens detected in goat and sheep fecal samples

Fecal samples obtained from small-scale goat and sheep farms in Tennessee and Georgia revealed the presence of *E. coli, Salmonella* spp.*, Shigella* spp.*, S. aureus*, and *S. saprophyticus*. A total number of 104 goats and 33 sheep from 30 farms were included in the analysis of this study. Presumptive pathogenic bacteria isolates identified by culture method were confirmed by PCR as described in Materials and Methods. Our findings displayed that *sdi*A and *vir*A genes were amplified for *Salmonella* spp. and, *Shigella* spp. while 16S rRNA genes were amplified for *S. aureus, E. coli, and S. saprophyticus*.

#### 3.1.1 *Salmonella* spp. in goats and sheep farms

Overall, the occurrence of *Salmonella* spp. in goat and sheep was notably low at 10% (3 out of 30) of the farms sampled in Tennessee (TN) and Georgia (GA) as shown in our study. The confirmation of *Salmonella* in the fecal samples was established through the amplification of *sdi*A gene, as depicted in **Figure 1**. Overall, the occurrence of *Salmonella* spp. in goat and sheep feces was 3.0%. Specifically, *Salmonella* spp. was detected in 2.2% of goats, which was higher than in sheep (0.8%) in the study areas. Noteworthy is the absence of *Salmonella* spp. in any of the goat fecal samples from Georgia.

#### 3.1.2 *Shigella* spp. in goats and sheep

Our results demonstrate that *Shigella* spp. was present in 63.3% (19 out of 30) of goat and sheep farms in TN and GA. **Figure 2** illustrates the amplification of the *vir*A gene in *Shigella* spp. isolates. The findings of this study reveal a collective prevalence of 35.0% of the pathogen in goats and sheep feces sampled from the farms. Specifically, 27.0% of goats and 8.0% of sheep tested positive for *Shigella* spp.

#### 3.1.3 *Staphylococcus aureus* in goats and sheep in feces

Confirmation of *S. aureus* presence in goat and sheep feces was achieved through the analysis of the 16S rRNA gene, as depicted in **Figure 3**. The pathogen was detected in 76.7% (23 out of 30) of farms across both states from which fecal samples were collected. *S. aureus* showed an overall prevalence of 91.3% in ruminants, with 56.4% of goats and 34.9% of sheep testing positive for *S. aureus* specifically.

#### 3.1.4 *Staphylococcus saprophyticus* in goats and sheep in feces

The presence of *S. saprophyticus* in the fecal samples was confirmed by amplifying the specific 16S rRNA gene, as depicted in **Figure 4**. In total, *S. saprophyticus* showed an overall prevalence of 81.0% in goat and sheep feces. More specifically, 60.0% of goats and 21.0% of sheep tested positive for *S. saprophyticus*.

#### 3.1.5 *Escherichia coli* in goats and sheep

**Figure 6:**
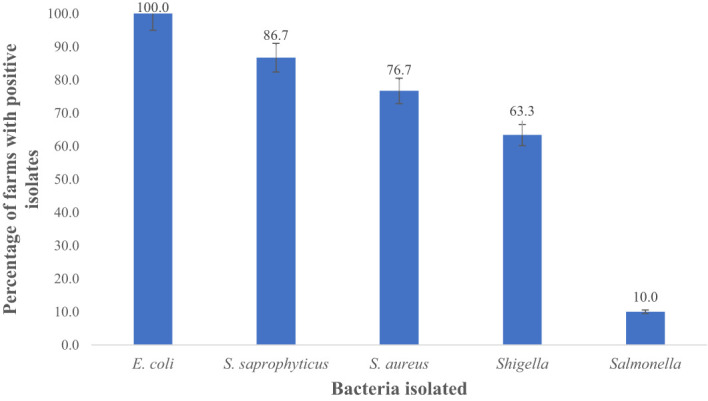
Total percentage of goat and sheep farms with prevalence of *E. coli*, *S. saprophyticus*, *S. sureus Shigella* and *Salmonella* in fecal samples. Number of farms positive for *E.coli =* 30*; S. saprophyticus* = 26; *S. sureus*= 23*; Shigella* = 19, *and Salmonella* = 3.

*E. coli* spp. was identified in fecal samples from both goat and sheep farms through the amplification of the 16S rRNA gene, as shown in **Figure 5**. Our results demonstrate that *E. coli* spp. was present in 100% (30 out of 30) of farms visited in TN and GA, as illustrated in **Figure 6**. The findings of this study reveal a collective prevalence of 94.9% for *E. coli* spp. in goat and sheep feces sampled from the farms. Specifically, 56.4% of goats and 38.5% of sheep tested positive for *E. coli* spp.

**Figure 7:**
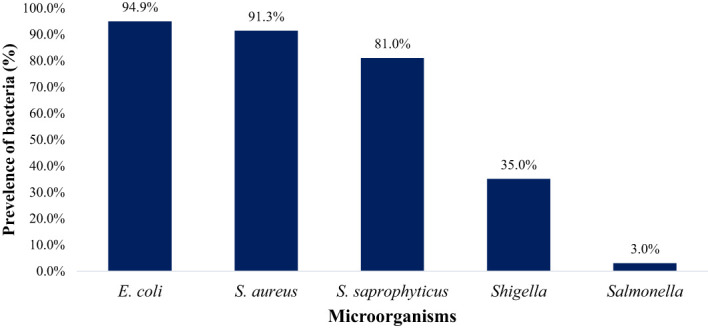
The overall prevalence of *E. coli*, *S. aureus*, *S. saprophyticus*, *Shigella* and *Salmonella* in goat and sheep fecal (TN and GA; Total samples =137).

In this study, 100%, 86.7%, 76.7%, 63.3%, and 10% farms were positive for *E. coli* spp., *S. saprophyticus, S. aureus*, *Shigella* spp., and *Salmonella* spp., respectively as shown in **Figure 6**. *E. coli* (94.9%) was notably higher (p < 0.05) compared to *S. aureus* (91.3%), *S. saprophyticus* (81.0%), *Shigella* (35.0%), and *Salmonella* (3.0%) as displayed in **Figure 7**.

### 3.2 Investigating antibiotic resistance across various antibiotic types and classes

**Figure 8:**
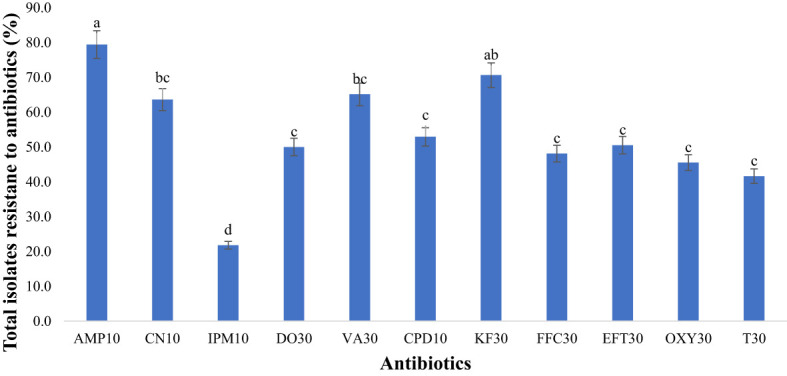
Antimicrobial resistance to antibiotics used in the study (n=418 isolatesfor each antibiotic). Antibiotics used: AMP10: Ampicillin; CN10: Gentamicin; IPM10: Imipenem; DO30: Doxycycline; VA30: Vancomycin; CPD10: Cefpodoxime; KF30: Cephalothin; FFC30: Florfenicol; EFT30: Ceftiofur; OXY30: Oxytetracycline; T30: Tetracycline). Statistical significance was tested using one-way ANOVA. The p-values (<0.05) were considered statistically significant. The letters (a–e) above the bars show significance of resistance within different antibiotics.

**Figure 9:**
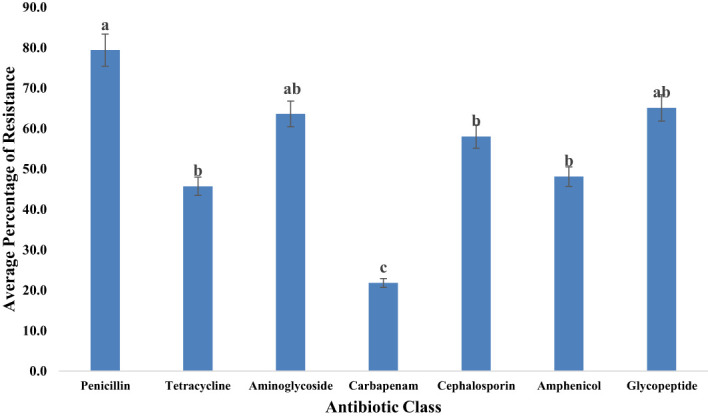
Average resistance (%) of all bacteria tested to each antibiotic class. Statistical significance was tested using one-way ANOVA. The p-values (<0.05) were considered statistically significant. The letters (a–e) above the bars show significance within groups.

Investigating antibiotic resistance across various antibiotic types and classes is crucial due to the growing concern surrounding antibiotic-resistant bacteria originating from food animals, which poses a significant threat to food safety. Therefore, this study aimed to examine the resistance of bacterial isolates to specific antibiotics (**Figure 8**) under selected antibiotic classes (**Figure 9**). It was also observed that most bacterial isolates exhibited multidrug resistance.

#### 3.2.1 Overall resistance of bacteria in goats and sheep fecal to a specific antibiotic

In our study, bacterial isolates from goats and sheep feces exhibited resistance to 11 antibiotics. Notably, there was significant resistance (p<0.05) to ampicillin, reaching 79.4% as depicted in **Figure 8**. Our study also unveiled notable resistance to cephalothin (70.6%), classified as a first-generation cephalosporin. Vancomycin exhibited high resistance at 65.1% in fecal samples. Gentamicin also showed considerable resistance, with a rate of 63.6%. Moderate resistance was observed with third-generation cephalosporins, including cefpodoxime (52.9%) and ceftiofur (50.5%). Among the tetracycline antibiotics, second-generation doxycycline displayed higher resistance (50.0%) compared to first-generation tetracyclines such as tetracycline (41.6%) and oxytetracycline (45.5%). Conversely, the lowest resistance was observed with imipenem at 21.8%.

#### 3.2.2 Resistance of bacteria isolates to different classes of antibiotics

Our investigation explored the resistance of bacterial isolates to various classes of antibiotics. (**Table 2**) displayed the different classless of antibiotics the bacterial isolates were resistant to. Among the identified bacteria, the penicillin class demonstrated the highest resistance level, reaching 79.4% as illustrated in **Figure 9**. Glycopeptides exhibited a notable resistance rate of 65.1% in our study. A significant resistance of 63.6% was observed for aminoglycosides. Moderate resistance (58.0%) to cephalosporins was also noted. Tetracyclines displayed a resistance rate of 45.7% among bacterial isolates. Additionally, our study highlighted the significant aspect of relatively low but noteworthy resistance (21.8%) observed in the carbapenem class.

**Table 2:** Antibiotics used regarding different classes used in this study.

Antibiotic class	Antibiotic agent
Aminoglycosides	Gentamicin
Glycopeptide	Vancomycin
Cephalosporin	Ceftiofur Cefpodoxime Cephalothin
Phenicol	Florfenicol
β-Lactam	Imipenem Ampicillin
Tetracycline	Doxycycline Oxytetracycline Tetracycline

## 4 Discussion

A total number of 104 goats and 33 sheep from 30 farms were included in the analysis of this study. Fecal samples obtained from small-scale goat and sheep farms in Tennessee and Georgia revealed the presence of *E. coli, Salmonella* spp.*, Shigella* spp.*, S. aureus*, and *S. saprophyticus.* Our results showed that *sdi*A and *vir*A genes were amplified for *Salmonella* spp. and *Shigella* spp. Additionally 16SrRNA genes were amplified in *S. aureus, S. saprophyticus*, and *E. coli*. *Salmonella* is widely recognized as a significant zoonotic pathogen, carrying economic implications for both animals and humans. The intestinal tract of a diverse array of domestic animals serves as the primary reservoir for *Salmonella*. Consequently, *Salmonella* from these animals may potentially contaminate various food products derived from animal sources, either directly or indirectly.

In the current study, the prevalence of *Salmonella* spp. (3%) in goat fecal samples was lower than that reported in other studies, such as 9.0% (Duffy et al., 2009) and 46.3% (Turkyilmaz et al., 2013). Our findings are consistent with those of Parker et al. (2020), who also reported the presence of *Salmonella* in sheep feces. The detection highlights that *Salmonella* spp., although an indicator of possible animal illness, can frequently be harbored by animals without outward signs of sickness, given its natural residence in their intestines and potential presence in their feces (Manishimwe et al., 2021). Microorganisms present in feces have the potential to easily contaminate various parts of an animal’s body, including its fur, as well as the immediate surroundings, such as pens, water, and soil. *Salmonella* infections in animals may present with symptoms such as diarrhea, lethargy, vomiting, or fever (CDC, 2022). Notably, humans can contract salmonellosis by handling animals, particularly through direct touch. Handling contaminated food and unintentionally transferring the bacterium from hands to mouths can lead to Salmonella infections. The minimal prevalence of *Salmonella* spp. of goat feces in Tennessee and its absence in Georgia goats suggest limited contamination. Nevertheless, our findings highlight the potential for the farming environment, including water and soil, to be contaminated with *Salmonella* spp. from goat feces.

Our research aligns with the findings of Li et al. (2022), who similarly identified the presence of *Shigella* spp. in both goats and sheep. The detection of *Shigella* spp. in farms raises concerns about the risk of shigellosis and gastrointestinal infections in food animals. While Shigella infections are commonly associated with human illness, documented cases indicate occurrences in goats and sheep as well (Ishlak et al., 2022). With the potential for zoonotic transmission, animals can serve as reservoirs and transmit pathogens to humans. Shigella infections in humans are often linked to the ingestion of contaminated food or water. However, documented evidence has revealed instances where human illnesses originate from exposure to Shigella-infected livestock or their excrement (Buskirk et al., 2022). This emphasizes an alternative pathway of transmission, highlighting the potential risk associated with direct or indirect contact with Shigella-contaminated animals or their environmental surroundings. Shigella transmission to humans can occur through direct or indirect contact with animals including goats and sheep, emphasizing the importance for individuals involved in livestock-related activities or exposed to livestock feces to take precautionary measures, such as wearing gloves and maintaining good hygiene.

In our study, *S. aureus* showed an overall prevalence of 91.3% in both goat and sheep feces. During slaughtering, this pathogen can contaminate carcasses, potentially transmitting to humans through the consumption of contaminated meat products. In a prior study by Tefera et al. (2019), this pathogen was also identified in 36.0% and 16.0% of sheep and goat carcasses, respectively. Dairy sheep and goat farms also incur substantial economic losses due to staphylococcal intramammary infections, with *S. aureus* recognized as the primary cause of clinical mastitis in small ruminants (Bergonier et al., 2003). A study by Widianingrum and Salasia (2021) reported a 53.3% prevalence of *S. aureus* in goats with subclinical mastitis. The repercussions of subclinical mastitis are noteworthy, encompassing substantial economic losses due to diminished milk quality and quantity, along with the associated expenses incurred in treating staphylococcal mastitis. It is also worth noting that Morel’s disease in both sheep and goats is associated with a subspecies of *S. aureus*, specifically, *S. aureus* subsp. *anaerobius*, with a predilection for affecting young animals, as explained by Haag et al. (2019). These findings underscore the importance of heightened vigilance and a comprehensive approach to addressing *S. aureus* in goat and sheep farms. Goats and sheep have the potential to act as reservoirs for *S. aureus*, thereby enabling the transmission of staphylococcal infections to humans.

Our findings are consistent with previous studies reporting the prevalence of *S. saprophyticus* in goats and sheep (Bader et al., 2021; Zandi et al., 2022). Additionally, *S. saprophyticus* has been documented in meat and milk samples, underscoring its widespread distribution and potential implications for food safety, as reported by Da Silva-Guedes et al. (2022). *Staphylococcus* spp. is acknowledged as a significant mastitis-inducing zoonotic pathogen, causing intramammary infections (IMIs) in small ruminants, including sheep and goats. Intramammary infections often take on a chronic character, resulting in reduced milk production, compromised milk quality, and an increased likelihood of progressing to clinical mastitis. The economic consequences of intramammary infections in small ruminants are substantial, encompassing significant costs related to both treatment and prevention (Kahinda, 2022).

In our study, the prevalence of *E. coli* (94.9%) was higher as compared to a study by Zaman and Sarker (2018), 20% of fecal samples from goats were found to be positive for *E. coli*. A previous study also documented the presence of *E. coli* in goats and sheep (Abreham et al., 2019). Given its natural occurrence in the gastrointestinal tract of mammals, the abundance of *E. coli* in fecal samples from goats and sheep is unsurprising. The contamination of food by *E. coli* is intricately associated with fecal contamination, particularly during slaughtering processes. This association is rooted in the prevalence of *E. coli* as the most found commensal enteric bacteria in both animals and humans. Furthermore, *E. coli* assumes a pivotal role as a zoonotic agent, posing a potential risk for infectious diseases in both animal and human populations (Costa et al., 2008). Bacteria of concern are increasingly infiltrating ecosystems and reaching consumers through meat products (Bartlett et al., 2013).

The dissemination of antibiotic-resistant foodborne pathogens and resistance genes can occur through environments contaminated with goat or sheep feces, which raises significant concerns for the safety of foods of animal origin. It is noteworthy that a notable percentage of bacterial isolates from goat and sheep feces in this study showed resistance to ampicillin. Ampicillin, a member of the β-lactams and a derivative of penicillin, is widely recognized for its broad-spectrum activity in combating bacterial infections. However, the extensive and longstanding use of penicillin, spanning over six decades, has played a role in the emergence of antimicrobial-resistant bacterial strains. In fact, the earliest identification of penicillin resistance dates to 1962 (Tran-Dien et al., 2018). Our study highlights that small-scale goats and sheep farms have bacteria that is resistant to ampicillin.

In this study, bacterial isolates from goat and sheep feces displayed a considerable resistance to gentamicin. Recent studies (Atlaw et al., 2022; Tanaka et al., 2023) have also highlighted the presence of gentamicin-resistant microorganisms in samples from goats and sheep. Gentamicin, an antibiotic commonly used to treat bacterial infections, particularly those caused by Gram-negative bacteria such as *E. coli*, has witnessed the emergence of resistance due to overuse and misuse. Gentamicin resistance not only poses risks to public health and animal welfare but also raises concerns about the possible transmission of resistance from animals to human food sources. This emphasizes the need for proactive measures to lessen its impact.

In our investigation, bacterial isolates from goats and sheep displayed the lowest resistance to imipenem. Imipenem, classified as a carbapenem, plays a crucial role as a last-resort antibiotic, particularly effective against microorganisms capable of producing extended-spectrum β-lactamases. Instances of carbapenem resistance have been documented in livestock, including goats and sheep in Southeast Asia, and Jordan (Khalifa et al., 2021; Obaidat et al., 2023). The resistance to imipenem raises significant concerns in healthcare settings, limiting the efficacy of this vital antibiotic in treating serious infections. The presence of antimicrobial-resistant bacteria resulting from the administration of antibiotics such as imipenem to goats and sheep poses a potential food safety hazard. These bacteria have the potential to persist in meat, milk, and other animal-derived food products. Failure to effectively eliminate these bacteria during food processing or cooking can lead to the transmission of antimicrobial-resistant strains to consumers. When bacteria exhibit resistance to imipenem, it significantly narrows the treatment options for human infections, particularly given its critical importance as an antibiotic. Consequently, this limitation in treatment alternatives may result in the development of more severe and challenging-to-manage infections in food animals.

In our study, second-generation doxycycline displayed higher resistance as compared to tetracycline and oxytetracycline, both belonging to the first-generation tetracycline class. The existence of tetracycline-resistant bacteria and resistant genes in animal fecal samples has also been confirmed by several studies (Turner et al., 2022; Osorio et al., 2023). Tetracycline, oxytetracycline, and doxycycline stand out as the most employed tetracyclines in veterinary medicine. The affordability and broad-spectrum nature of tetracyclines have contributed to their extensive utilization in various applications.

Vancomycin demonstrated high resistance to bacteria isolates from goat and sheep feces in the current study. The presence of vancomycin-resistant enterococcus in livestock has been documented in several prior studies (Zaher et al., 2023). In animal health, vancomycin is frequently used to treat bacterial infections in livestock, especially when conventional antibacterial agents are ineffective (Mutua et al., 2020). However, the use of vancomycin in animal husbandry has raised significant concerns due to its potential contribution to the emergence of antibiotic resistance. The vancomycin resistance observed in our study in goat and sheep fecal samples can potentially transfer from these animals to farms and humans. Such transmission of resistant bacteria from food animals to humans can ultimately lead to a decline in human health. Widely used in both veterinary and human healthcare, vancomycin is primarily employed for managing infections caused by Gram-positive microorganisms, particularly methicillin-resistant *S. aureus* (MRSA) (Nandhini et al., 2022).

In our investigation, bacterial isolates from goat and sheep feces displayed resistance of Gram-negative bacteria to third generation cephalosporins. This resistance encompassed cefpodoxime and ceftiofur. Additionally, our study unveiled notable resistance to cephalothin classified as a first-generation cephalosporin. Recent studies have also reported a substantial prevalence of cephalothin-resistant bacteria in animal production (Khong et al., 2023; Obeng-Nkrumah et al., 2023). Although the sensitivity of third generation cephalothin is diminished in Gram-positive bacteria, an elevated sensitivity is observed in Gram-negative bacteria (Bui and Preuss, 2023). Despite this, cephalothin remains a preferable antibiotic choice against Gram-positive bacteria. In a previous study (Herawati et al., 2023), Gram-negative bacteria, including *Salmonella* and *E. coli*, isolated from goats and sheep, were found to display resistance to ampicillin, tetracycline, cephalothin, and gentamycin.

According to our results, a moderate resistance was observed in the case of florfenicol. Florfenicol, which is only used to treat animal infections, is a derivative of chloramphenicol that is active against chloramphenicol-resistant isolates (Apley, 2015). The challenge of florfenicol resistance is escalating, with an increasing prevalence of drug-resistant bacteria in animals and breeding environments (Li et al., 2020). Elevated levels of resistance may impede the effectiveness of florfenicol in treating infections, potentially necessitating alternative antibiotics or treatment strategies. Recognizing and monitoring florfenicol resistance is crucial to ensure the effective treatment of bacterial infections in animals and prevent the spread of resistant strains.

In our study, bacterial isolates displayed resistance to multiple antibiotic classless and penicillin displayed the highest resistance. As noted by Sivagami et al. (2020), diverse pathogens have developed resistance to different antibiotic classes, posing a significant threat to animal, human, and environmental health. The resistance to penicillin and other antibiotics in the penicillin class, such as amoxicillin and ampicillin, raises significant public health concerns. Penicillin is extensively used in food-animal farming systems in the USA and globally, contributing to substantial resistance that has developed over an extended period of use. The overuse and misuse of penicillin and other antibiotics in both animal production and human health have contributed to the emergence of penicillin-resistant bacteria, particularly among Gram-positive bacteria like *Streptococcus pneumoniae* and *Staphylococcus aureus* (Mancuso et al., 2021). There is a need for careful management of antibiotic use in sheep and goat farms to mitigate the development of antibiotic resistance and its potential impact on both animal and human health.

Resistance to tetracyclines was also observed among bacterial isolates, aligning with the findings of Mohiuddin et al. (2020); and Smoglica et al. (2022). These studies indicated the presence of tetracycline-resistant pathogens in the feces of goats and sheep. Tetracyclines are extensively used in veterinary medicine to prevent and treat a broad spectrum of bacterial infections. Resistance to these antibiotics carries significant implications for public health, animal health, and the effectiveness of antibiotic treatments. Tetracycline has been widely employed for preventing and treating various bacterial infections in animals, including goats and sheep (Mala et al., 2021 However, concerns have arisen due to the overuse and misuse of tetracyclines in veterinary medicine, leading to the presence of antibiotic residues and the development of resistance in various environmental components (Xu et al., 2021). The use of tetracycline in animal agriculture has played a significant role in the development of tetracycline resistance in bacteria transmitted to humans through the food chain (Manyi-Loh et al., 2018).). Infections resistant to tetracycline have become more challenging to treat, necessitating the use of more potent and expensive antibiotics.

Bacterial isolates in the current study demonstrated a significant resistance to aminoglycosides. In veterinary medicine, particularly in goats and sheep, concerns have been raised regarding the resistance to aminoglycosides, which include medications such as gentamicin, amikacin, and streptomycin. This has significant clinical implications, as aminoglycosides are commonly employed to treat various bacterial infections in goats and sheep, and resistance can markedly restrict available treatment options.

Although, carbapenems are not licensed in livestock or veterinary fields and are not commonly tested in animals (Ramírez-Castillo et al., 2023), our study underscored the noteworthy aspect of bacteria isolates resistance to the carbapenem. The persistence and dissemination of carbapenem-resistant bacteria in the environment pose significant and interconnected risks to the health of goats, sheep, and the farm environment. Despite the low resistance observed in bacteria isolates in this study, the prevalence of Carbapenem-resistant Enterobacteriaceae (CRE) and other resistant Gram-negative bacteria is on the rise, posing a substantial threat to both human and animal health. Carbapenems are acknowledged as one of the most potent classes of antibiotics, typically reserved as a last-line treatment option for severe bacterial infections. They exhibit broad-spectrum activity, proving more effective than other β-lactams, especially against resistant bacteria (Aurilio et al., 2022).

Resistance of bacterial isolates from goat and sheep feces to cephalosporins was displayed in this study. Cephalosporin-resistant strains within livestock and agricultural settings are of particular concern and may compromise animal health, leading to decreased productivity and necessitating the employment of alternative therapeutic agents. A previous study suggested that these *E. coli* strains, and their antibiotic resistance genes, can spread from food-producing animals, via the food-chain, to humans (de Been et al., 2014). The dissemination of antibiotic-resistant bacteria in environmental matrices presents a multifaceted threat with far-reaching implications for both animal agriculture and human health.

Resistance to the amphenicol class, encompassing florfenicol and chloramphenicol, was observed in bacterial isolates within our study. This resistance highlights concerns regarding the efficacy of bacterial infection treatments in goats and sheep, necessitating a reevaluation of treatment approaches and exploration of alternative antibiotics. Amphenicols, a group of broad-spectrum antibiotics primarily utilized in veterinary medicine, are employed for treating illnesses in agricultural animals (Trif et al., 2023). Notably, amphenicols, including chloramphenicol and florfenicol, are extensively utilized in livestock farming, leading to their accumulation in animals and posing risks to consumers (Wu et al., 2021). Moreover, chloramphenicol exhibits high toxicity towards the human hematopoietic system, potentially inducing adverse effects such as aplastic anemia (Cerutti et al., 2021).

In our study, bacteria isolated displayed notable resistance to glycopeptide antibiotic class. It’s crucial to emphasize that the off-label use of these medications is strictly prohibited, even if the conditions for such use are met. Vancomycin, a prominent glycopeptide, serves as a vital defense against various drug-resistant bacteria, particularly methicillin-resistant *S. aureus*. However, the widespread transmission of vancomycin-resistant genes among animals, humans, and bacteria poses a significant challenge (Aqib and Alsayeqh, 2022). In the United States, the off-label use of glycopeptides is banned in all food-producing animal species. The presence of vancomycin-resistant bacteria in goat and sheep feces raises concerns, especially more recent variant of MRSA has shown to demonstrate anti-glycopeptide drug resistance.

## 5 Conclusion

In summary, the identification of antibiotic-resistant *E. coli* spp.*, Salmonella* spp.*, Shigella* spp.*, S. aureus*, and *S. saprophyticus* in feces from small-scale goat and sheep farms highlights the concerning prevalence of antimicrobial resistance in agricultural settings. Resistant bacteria in goats and sheep can disseminate throughout the farm environment, including soil and water, posing risks to ecosystems and the safety of food products derived from these animals. Additionally, with the rising popularity of goat and sheep meat dishes, consumers may face an elevated risk of infection with antibiotic-resistant bacteria. Although our fecal sample analysis was limited in scope, our data underscores the potential for feces from small-scale goat and sheep to act as reservoirs for multidrug-resistant bacteria. The resistance of bacteria in food animals to antibiotics further complicates the landscape of food safety.

## Data availability statement

The original contributions presented in the study are included in the article/supplementary material. Further inquiries can be directed to the corresponding author.

## Author contributions

AB: Conceptualization, Data curation, Formal analysis, Investigation, Methodology, Resources, Software, Supervision, Validation, Visualization, Writing – original draft, Writing – review & editing. AK: Conceptualization, Data curation, Formal analysis, Funding acquisition, Investigation, Methodology, Project administration, Resources, Supervision, Validation, Visualization, Writing – review & editing.
